# Exploring factors that shaped Syrian refugees integration into Lebanon’s national health system using Kingdon’s Multiple Streams Framework

**DOI:** 10.1186/s13031-025-00680-2

**Published:** 2025-07-10

**Authors:** Fadi El-Jardali, Gladys Honein-AbouHaidar, Lama Bou-Karroum, Sabine Salameh, Sarah E. Parkinson, Rima Majed

**Affiliations:** 1 Department of Health Management and Policy, Faculty of Health Sciences, American University of Beirut, Beirut, Lebanon; 2 Knowledge to Policy (K2P) Center, American University of Beirut, Beirut, Lebanon; 3 Department of Health Research Methods, Evidence, and Impact (HEI), McMaster University, Hamilton, ON Canada; 4 Hariri School of Nursing, American University of Beirut, Beirut, Lebanon; 5 Johns Hopkins University, Baltimore, MD USA; 6 Department of Sociology, Anthropology & Media Studies, American University of Beirut, Beirut, Lebanon

**Keywords:** Refugees, Health system, Lebanon, Policy analysis

## Abstract

**Background:**

Since the start of the Syrian conflict in 2011, neighboring country Lebanon has hosted the largest number of refugees per capita in the world. To meet refugees’ health care needs, Lebanon adopted an integrated model of care. This paper explores the key factors and events that have shaped the policy on the integration of Syrian refugees into the Lebanese national health system through a policy analysis.

**Methods:**

The research team adopted a qualitative approach that employed in-depth interviews with 12 key informants (2 ministers, 4 non-governmental organizations, 3 advocacy group representatives, and 3 healthcare managers) and document review. Thematic framework analysis was used to analyze the data guided by the Kingdon’s Multiple Streams Framework.

**Results:**

Problem factors that influenced Lebanese health policy towards Syrian refugees include the sheer number of refugees with urgent health care needs who entered a fragile, highly privatized health care system, and political and sectarian dissension around the refugee issue, both of which contributed to a slow government response. In the policy stream, international non-governmental organizations concerned with refugee health started to engage with local authorities. In December 2014, the Lebanon Crisis Response Plan strategy was issued by the government and various partners that iterated the strategy to respond to Syrian refugees’ needs. Under the political stream, Lebanon’s historical experience with Palestinian refugees, and specifically concerns regarding fear of domiciliation, influenced the unofficial implementation of a ‘no camp policy’ strategy at the onset of the crisis, which in turn shaped healthcare integration. Further, international non-governmental organizations joined efforts to fund and supplement health care services, while think tank policy organizations advocated for refugees right to healthcare and host community support.

**Conclusion:**

This study highlights the role of global actors, such as UNHCR, WHO among others, as the main entrepreneurs in integrating refugees into the Lebanese health care system. It also underscored the ad-hoc non-systematic approach with which the policies around refugee health response were made in Lebanon and the influence of political factors. Although the mutual benefits to both host and refugee communities were recognized, many challenges threaten integration, foremost among them the model’s financial sustainability.

**Supplementary Information:**

The online version contains supplementary material available at 10.1186/s13031-025-00680-2.

## Introduction

At the end of 2022, 117.2 million individuals worldwide were forcibly displaced, 72% were hosted in neighboring countries as refugees [1]. Camp-based accommodation has become increasingly unsustainable due to the sheer number of refugees [2], the duration of displacement (refugees, on average, maintain their status for 10.3 years before repatriating or resettling) [3], and living conditions that expose them to systematic violations of their basic human rights [4]. Hence, refugees are increasingly housed within host communities. According to the United Nations Health Refugee Commission (UNHCR), integration of refugees is a “multifaceted two-way process” which requires readiness and efforts from both host and refugees to meet all needs of the refugee by the former and to adapt to the host society by the latter [5]. Integration is complex and multilevel, comprising economic, social, cultural and health integration.

Historically, refugee health care has been provided primarily through dedicated, camp-based health clinics managed by the United Nations High Commissioner for Refugees (UNHCR), the United Nations Relief and Works Agency for Palestine Refugees in the Near East (UNRWA) (for Palestinian refugees), or international non-governmental organizations (INGOs). Given that patterns of refugee settlement are changing, this parallel health service system has become unsustainable. Instead, states and humanitarian actors are increasingly adopting an integrated model that serves refugees through national health care systems. The UNHCR has called for the integration of health services for refugees within national health systems to enable refugees and surrounding host populations to access similar services, and avoiding parallel systems, when possible [6].

Emerging evidence underscores host country governments’ resistance to integration; states often portray refugee crises as short term and argue that they may lead to over-stretching of limited resources for national populations [7]. The World Bank consequently established a US$2 billion fund for host governments to support refugees’ integration into national healthcare systems [8]. The international community thus expects that host countries’ will make their national health care systems accessible to refugee populations. This support is particularly important in countries where the resources are scarce and where host communities already face differential access to services based on affordability, availability and accessibility [9].

A number of studies explores the intersection of political, economic, and social factors that shape refugees’ experiences of national healthcare systems access in host countries particularly in low and middle-income countries (LMICs) [10–12]. In Lebanon, researchers have identified various practices and policies that shape refugees’ experiences of the national healthcare system and other provider networks, including those linked to security, geographic location, host community dynamics, economics, and status as “cross-border” migrants [13–15]. Our research explores key factors and events that shaped the policy on the integration of refugees into the Lebanese health system through a policy analysis using Kingdon’s Multiple Streams Framework. Lessons learned from this study can inform the design and implementation of policies for achieving universal health coverage in Lebanon.

### Country setting

**Fig. 1:**
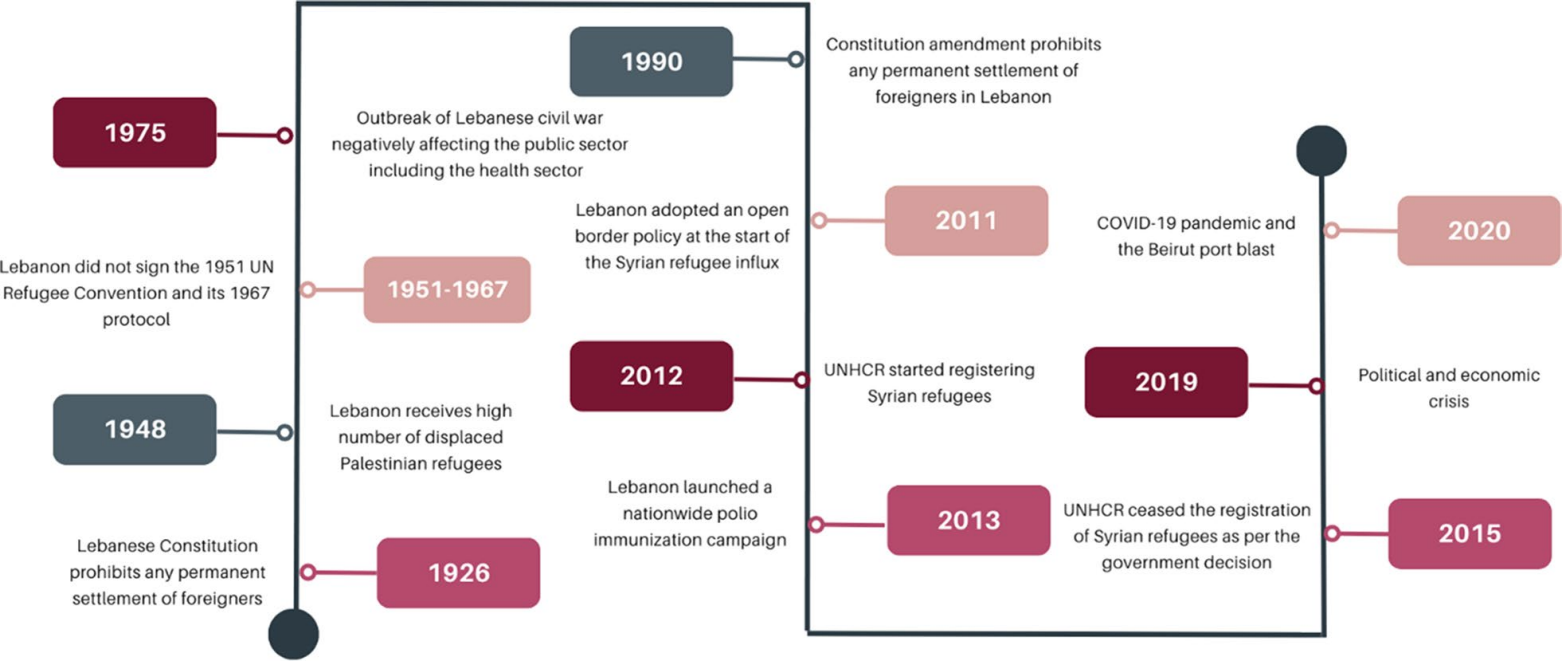
Timeline of key events and policies shaping the health refugee response 1926–2020

Lebanon has a long history of hosting displaced populations, despite its often unstable political situation and its generally poor infrastructure (Fig. 1). The Lebanese Constitution of 1926 (and its amendment in 1990) prohibits any permanent settlement of foreigners [16, 17]. In addition, Lebanon is not a signatory of 1951 UN Convention Relating to the Status of Refugees and its 1967 Protocol, meaning that Lebanon cannot be a permanent country of asylum [18, 19]. Thus, Lebanon considers fleeing populations as displaced and not refugees.

In 1948, Lebanon received a high number of displaced Palestinians, who settled in impoverished camps as well as among the Lebanese host population [20]. The camps in particular became central sites of Palestinian nationalist and leftist mobilization [17]. Palestinian refugees received camp-based primary health services, through a parallel system, delivered and financed by UNRWA and supported by the Palestinian Red Crescent Society (PRCS) [21–23]. The PRCS also provided affordable healthcare to poor Lebanese [20].

Lebanon is also a country with a long history of political instability. The 1975–1990 civil war, and the political conflicts thereafter contributed to the weakening of the public sector and the country’s economy. Lebanon’s economy was already strained with a debt to Gross Domestic Product ratio of over 180% [24]. These political and economic challenges, in addition to the proliferation of a system of non-state sectarian welfare providers [25–27], undermined the government’s provision of social protection to its own citizens including health coverage. Humanitarian aid by United Nation (UN) agencies and INGOs has supported Lebanon all along since the civil war and was involved in responding to the needs of the Palestinian and recently Iraqi refugees [24].

Syria neighbors Lebanon to the east and north. Since 2011, millions of Syrians have fled into Lebanon. Syrian refugees found shelter in local communities; 22% lived in informal tented settlements mostly in east and north of the country while others dwelled among host communities across the country [28]. According to recent UNHCR figures, 88% of the Syrian refugees in Lebanon are living in extreme poverty and 34% have challenges accessing healthcare [28]. The Lebanese government officially estimates the number of Syrian refugees in Lebanon to be approximately 1.5 million, however the actual number of Syrian refugees in Lebanon is unknown. From 2011 until mid-2015, many refugees crossing the border to Lebanon were registered by UNHCR as refugees, and are consequently referred to as “registered refugees”. In May 2015, the Government of Lebanon halted Syrian refugee registration. UNHCR continued to record the number of newcomers, who are referred to as “recorded refugees”. An unidentified number of refugees crossed the border to Lebanon and were live without official registration or recording, those are referred to as “unregistered” [29]. Further, since 2011, only an estimated 31% of newborns in Lebanon were registered [28].

Since 2019, Lebanon has been facing a multifaceted crisis: an economic collapse considered to be one of the worst in modern history, the COVID-19 pandemic, and the August 4, 2020 Beirut port blast which physically destroyed much of East Beirut, killed 218 and wounded over 7,000 people, rendered most of the city’s health care institutions inoperable, displaced 300,000 people, and caused US$390–475 million worth of damages [30]. These events occurred in the context of a protracted refugee crisis that made Lebanon the host of the highest number of refugee per capita worldwide [31].

### Kingdon’s multiple streams framework

**Fig. 2:**
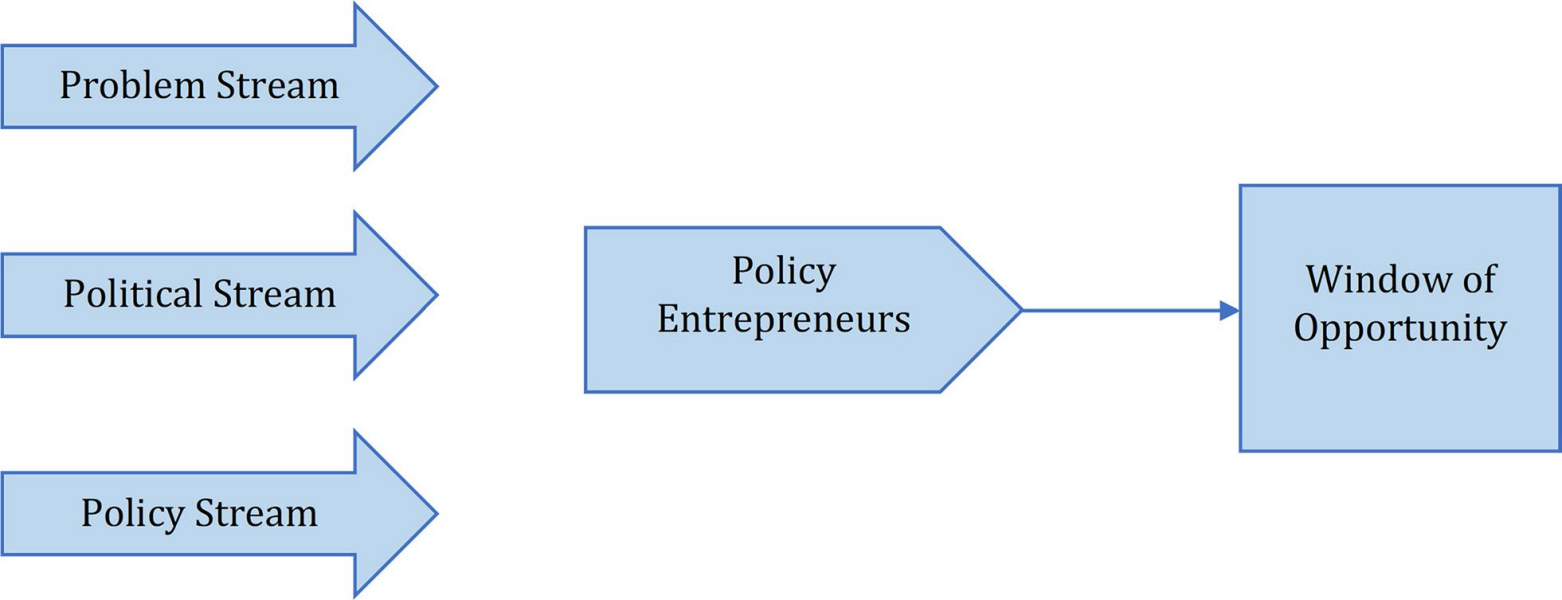
The Multiple Streams Theory of Policy Change [33]

This study adopts the Kingdon’s Multiple Streams Framework as conceptual model that enables us to examine the steps in formulating the policies related to Syrian refugees’ access to health care in Lebanon. According to Kingdon, there are three streams (problem stream, policy stream and political stream) that operate separately and independently but their convergence creates a window of opportunity mediated by policy entrepreneurs leading to setting up the policy agenda [32] (Fig. 2). In the presence of this opportunity, the policy entrepreneurs need to act quickly on the policy.

## Methodology

### Study design

This study is based on a qualitative research approach triangulating two data types: document review and in-depth interviews. The document review aimed at examining the key policies that have shaped health integration policy, adoption, and implementation at the structural, institutional and individual/community levels. The in-depth interviews aimed at gathering the key stakeholders’ viewpoints on a range of issues related to the health integration policy. Triangulation increased the rigor of the findings, reduced bias, and corroborated findings across two different data sources [34]. We adopted the consolidated criteria for reporting qualitative research (COREQ) [35].

### Document review method

#### Sampling

We reviewed 284 documents including: (1) peer reviewed articles published before October 2020 using OVID Medline, PubMed, and Scopus (Additional file 1), (2) reports, strategies, plans and policies from governments, national, and international reports and reference list searches (Additional file 2) and (3) news media articles collected from the press tracing conducted by the Knowledge to Policy (K2P) Center at the American University of Beirut. K2P Press Tracing encompasses all health-related stories and issues reported in seven Lebanese and regional newspapers published since 2010.

#### Data management

Each document retrieved was reviewed and summarized in a data collection matrix guided by Kingdon’s multiple stream model. The matrix was first piloted on a set of 20 documents where the review of documents was conducted independently by two different reviewers from the research team to cross-check information. The results were then presented in a webinar to a number of experts from a multidisciplinary background and revised according to the feedback received.

#### Data analysis

Analysis was done based on a qualitative approach to analysis, i.e. excluding the quantification typical of conventional content analysis [36]. At first, we reviewed each document and identified meaningful and pertinent information relevant to the central question of the research. We organized the information targeting the central question of the research [37].

### In-depth interviews

We used semi-structured in-depth interviews to gather the thought processes and viewpoints of key stakeholders involved in the formulation of healthcare policies regarding Syrian refugees in Lebanon.

#### Sampling

We adopted a non-probability, purposive sampling approach with maximum variation. We purposefully targeted policy and decision makers, e.g. ministers of health, heads of departments, patient rights organization (advocacy groups), and international actors such as representatives from UNHCR and the United Nations Children’s Fund (UNICEF). We also targeted decision-makers and health care managers from regions with varied refugee and host population characteristics (proximity to borders, urban, rural). In total, our sample included 12 key informants: 2 ministers, 4 non-governmental organizations, 3 advocacy groups, and 3 health care managers.

#### Recruitment

We sent an email invitation (Additional file 3) to a list of potential policy and decision makers from the different entities iterated in our sampling. In the invitation, we explained the purpose of the study, explained the data collection approach, and solicited their approval to participate. Those who consented were contacted by email to set a date, time, and location of convenience to conduct the in-depth virtual or face to-face interviews. We collected data until we reached saturation that is after the point where no new data is emerging.

### Data collection

#### Interview guide

We used a semi-structured interview guide to pose key questions framed by the three constructs of the Kingdon’s multi-stream model (problem, policy, and politics). Each question was followed by probes to elicit more detailed and elaborate responses to those key questions (Additional file 4—Interview guide).

#### Data collection process

The data was collected virtually or face-to-face between September 2021 and May 2022. Two interviewers (SS & LBK) led the discussion in Arabic. After introducing herself, the interviewer went over the consent form including the purpose of the study, the rationale for inviting participation, and the data collection process. She also iterated that the participation is voluntary and that they can skip questions or withdraw from the study at any time without consequences. At the end, she asked for permission to proceed with the questions and for recording the discussion. Once approved, the audio-recording started. For participants who did not agree to be audio-recorded, notes were taken.

The interviewer started asking the introductory question followed by a key question (problem stream). Thereafter, there was no order for asking the questions, but rather the interviewer was following the participants’ lead. The interviewer was actively listening, and constantly choosing the best probing approach to elicit information from the participant, while remaining neutral to any interpretation. Interviews lasted between 45–60 min on average.

#### Data management and analysis

The audio-recording was translated and transcribed by SS. LBK validated the translation by randomly selecting transcripts and back translating them to ensure preserving the meaning between the original and translated data. Noteworthy to indicate that both SS and LBK share the same language and culture of participants, important to maintain the integrity of the translation process [38].

Two data coders conducted the analysis (GHA and SS). To protect their privacy, participants were coded as PM (policy and manager) and were given a serial number ranging from 01 to 12. Quirkos software was used for the data analysis [39].

The analytical approach was the thematic framework by Crabtree and Miller [40] detailed in Additional file 5. Framework analysis operates from a pragmatic epistemology. It is divided into two elements: First, creating an analytic framework, and second applying this analytic framework. The following five steps define this approach: *(1) data familiarization; (2) identifying a thematic framework; (3) indexing all study data against the framework; (4) charting to summarize the indexed data; and (5) mapping and interpretation of patterns found within the charts* [41].

### Ethics

Verbal or written consent for participation and audio-recording were provided before interviews commenced. Participant quotations and organisations were de-identified to fulfill the governing ethics agreements. Ethical approval was granted by the American University of Beirut Human Ethical Review Board (SBS-2019-0287).

## Results

The results section presents a synthesis of qualitative findings from the document review and interviews categorized by the three streams of the Kingdom Model and the emerging sub-themes (Table 1).

**Table 1:** Themes and subthemes

Stream	Sub-theme	Exemplary quotes
Problem stream	Influx of refugees of an epic proportion	*‘Due to the Syrian refugee crisis, we had a huge increase in demand for the healthcare services and we already know that our Lebanese health sector is not solid enough’ PM-06* *‘Our government faced with this huge influx of refugees couldn’t adjust to this situation without resorting to the help of organizations, municipalities, and the private sector’ PM-09*
	Syrian health profile and health care needs	*‘The Lebanese health system assumed responsibility and attended to the Syrian refugee health needs’ PM-02*
	Frail and highly privatized health care system	*‘Maybe we should start talking about the country’s background. Our Lebanese health system was never at its best, … With the influx of Syrian refugees, it wasn’t easy for the health system to respond quickly and adequately to these refugees’ medical and non-medical need’ PM-10*
	Political and sectarian tensions between Lebanese and Syrian communities	*‘After 2015, the borders closed because the government realized that we are reaching a phase where the number of refugees became alarming. So, they approached the matter from a demographic perspective. As, they considered that the numbers of refugees could lead to a huge demographic change in the Lebanese already very shaky structure’ PM-10*
	Lebanese Government did not act swiftly, compounding the stress on the health care system	*‘The Lebanese government was in the back seat… the government was not taking the lead to deal with this [refugee] issue* *PM-12*
Policy stream	INGO creating a fragmented parallel system	*‘For hospital admissions, we did create kind of a parallel system with this private insurance scheme covering Syrian refugees. Which is very similar to the one adopted for the Palestinian refugees’ PM-01*
	The timid beginning for policies started with the polio outbreak	*‘I remember that when it came to vaccination campaigns for polio and other vaccinations, we established a system between MOSA and MoPH on how to include the refugees in the vaccination campaigns because if only the Lebanese were vaccinated without including the Syrian refugees it would have been useless’PM-12*
	Lebanon Crisis Response Plan (LCRP) 2015–2017	*‘Syrian Refugees were one of the key components of LCRP response since 2014, since the crisis started in Lebanon’ PM-06*
	Shape of the health integration policy in Lebanon	*‘Beneficiaries form all nationalities can access the PHCs [primary healthcare centers], without the need for any type of identification. Also, they only pay an amount of 3000 L.L. per consultation and they can consult a general practitioner or a specialist’ PM-05* *Their integration is due to the fact that they had to be offered secondary health services either by private or public hospitals. Private hospitals didn’t want to receive the Syrian refugees because this meant they will be reimbursed the MoPH’s tariffs. So, the peripheral private hospitals and public hospitals took the initiative and received these refugees’ PM-02*
Political stream	'No camp policy' adopted at the onset of the crisis	*‘I believe the adoption of a no camp policy is a purely political decision. For the government, they believed this crisis could last and it could affect the country’s demographic structure as well as change the religious sects’ numbers’ PM-10*
	Previous Palestinian refugee experience	*‘The fact that there is no formal recognition of the Informal Tented Settlement is definitely related to the existence of those Palestinian camps. I think there was a political instrumentalization of what happens with the previous camps experience and this definitely informed the attitude of certain political leaders’ PM-01*
	Policy think-tank organizations advocating on behalf of the refugees	*‘I think, the advantage we have at the institute is that we are a research and policy institute. So, we try to produce first hand data from our research on the ground and our field work, whether on our own or with partners. I believe, the edge we have is that we have a very strong convening power and that we’re able to bring stakeholders and policy makers around the table to discuss our work to come out with a certain policy’ PM-04*
	The community played a role in ensuring the implementation of health integration policy	*‘The local community played an important role at the beginning of the crisis, for example “schools in Tripoli, North of Lebanon, enrolled around 10,000–12,000 Syrian refugee children’ PM-12*

### Problem stream: several stressors set the stage for the health integration policy

The problem factors that influenced the policy were the sheer number of refugees and their urgent health care needs taking place in a frail and highly privatized health care system and in the midst of political and sectarian dissension around the refugee crisis, both of which led to a slow government response.

#### Influx of refugees of an epic proportion

At the onset of the crisis, Lebanon kept its border open until 2014 to all Syrian refugees though many still crossed illicitly due to fear of state authorities [42]. At the end of 2014, the number of registered Syrian refugees reached 1.1 million constituting 25% of the Lebanese population, the highest per capita in the world [43]. The impact of this refugee influx, created a tangible stress on the health care system across all the sectors as indicated by most participants and reflected in this quote: *‘With the influx of Syrian refugees, it wasn’t easy for the health system to respond quickly and adequately to the refugees’ medical and non-medical needs’ PM-10.*

In light of the large size of the displaced population and the protracted aspect of the crisis, the Lebanese government started putting more restrictions on the entry of Syrian refugees at the end of 2014 [18, 44]. As per government instructions, the UNHCR suspended the registration of displaced Syrians in 2015. Hence, many became residents of Lebanon without registration, which eventually have had repercussions on their access to health services: *‘UNHCR registration has stopped because of the government long time ago in 2015. In case they are registered they would be able to benefit from services and support’ PM-04.*

#### Syrian health profile and health care needs

Around 64% of the Syrian refugees’ population has at least one member with a specific health need including physical or mental disability, chronic illness, temporary illness or injury, a serious medical condition, and/or needing support in basic daily activities [45]. The refugee population was also characterized by high prevalence of chronic diseases [45]. The majority of all Syrians registered by UNHCR are women and children, with more than half (54%) of the refugee population being under the age of 18, 2% of whom have a disability [45, 46]. The rates of caesarean sections and the needs of antenatal care are also high among displaced Syrians while family planning use is low [47]. Sexual and gender-based violence and mental health problems have also increased as a result of displacement [48]. These health needs have strained the Lebanese health system [49] but, as one participant indicated that the Lebanese health care system was *‘able to absorb the refugees’ influx the only problem was straining the system PM-08.*

#### Frail and highly privatized health care system

Syrian refugees settled in a country where the health care system is historically highly privatized and frail. The history of conflict and political instability, specifically between 1975 and 1990, contributed to the weakening of the public health sector [25, 26, 50] and the outgrowth of the private sector and nongovernmental organizations (NGOs).

Decision-makers concurred that the public health care system was operating *at a loss even before the onset of the crisis*. For instance, one policymaker mentioned that *‘a lot of the public hospitals were already in distress financially, logistically, *etc*.’ PM-02.* Another policymaker added *‘Our Lebanese health system was never at its best, … With the influx of Syrian refugees, it wasn’t easy for the health system to respond quickly and adequately to these refugees’ medical and non-medical need’ PM-10.*

The Lebanese health system is a public–private mix model [51]. Primary health care services, 68% of them are governed and funded by NGOs and under contract with the Ministry of Public Health. Secondary and tertiary services were and still are 80% private [21, 50] as they are characterized by high out-of-pocket expenditure. Hence, accessibility to the host community is governed by affordability [42], refugees are at even further disadvantage as this decision-maker indicated:

*‘I think that it is related to the fact that we’re living in a highly privatized system, and that as soon as you are vulnerable your access is being jeopardized. In that regard, I think that being a refugee makes you even more vulnerable’PM-01.*

#### Longstanding lebanese-syrian tensions and their influence on policy debates

It is noteworthy to indicate that tensions between Lebanese and Syrians existed long before the Syrian war. For more than 30 years, Syria’s military presence in Lebanon, beginning with its intervention in the 1975–1990 Lebanese Civil War and support of armed Lebanese and Palestinian political parties, fomented political unrest and polarization. The occupation facilitated cross-border migration for Syrian laborers, over half a million of whom worked in Lebanon, mostly at very low wages [52]. The assassination of former Prime Minister Rafiq Hariri in 2005 exacerbated this tension, leading to a popular uprising, counter protests, and the abrupt end of the Syrian military presence in the country [53].

The onset of the Syrian crisis amplified these existent frictions, which led to a political divide within Lebanese politics and stirred up tension in the ministerial cabinet regarding parties’ stance vis-à-vis the Syrian regime. Some politicians advocated against hosting refugees: *At the beginning, there was a lot of hesitation whether the Lebanese government should take care of the Syrian refugees or not. There were two different opinions within the government, one opinion was to close the Lebanese-Syrian borders to avoid dealing with and receiving the refugees, the other opinion was that this is a humanitarian cause’ PM-12.*

Most refugees were concentrated in underserved, poor, and vulnerable host communities, especially in North Lebanon and the Beqaa region. They shared scarce resources and poor public health infrastructure with the host community. Competition over jobs, rising unemployment among Lebanese, and the economic recession in Lebanon increased the hostility between the host and the refugee population [42]. Growing resentment stemmed from the perception of refugees as a source of the economic deterioration and competition over resources, employment, and services. As per the World Bank, GDP growth decreased from 8% in 2010 to 2.5% in 2014 [53], while unemployment increased from 6.8% to 8.7% [54] which some attributed to the massive influx of Syrian refugees [55]. Additionally, political and media figures often framed Syrian refugees as being a threat to Lebanese culture and identity, specifically due to the perceived risk they posed to Lebanon’s confessional balance [52]. This was echoed by one participant: *“I believe that they have competed with the host community for the national resources, especially that the funds weren’t allocated to the national health system due to the lack of trust in the government’ PM-07.*

#### Lebanese government did not act swiftly compounding the stress on the health care system

In principle, a crisis of such a magnitude would prompt any government to act swiftly and with agility to issue a policy response to the crisis. Instead, at the beginning of the Syrian influx into Lebanon from 2011–2013, the Lebanese government was evasive and opposing an emergency response for several reasons. The position in this initial stage centered on *‘*self-distancing’ *(PM-07)*, verging to *‘refusing to provide support’ (PM-04)* out of ‘*fear of domiciliation’’* (*PM04)*.

There was a *dissension* among the Council of Ministers on whether the Lebanese Government should provide the necessary health care to Syrian refugees, which had led to a gridlock and inertia.

*The Council of Ministers was not able to make drastic decisions to deal with the Syrian refugees’ PM-12’*

Those opposing support for the Syrian refugees even resisted the initial support from international non-governmental organizations.

*‘During the early stages of the crisis we faced problems with UNHCR, first the Ministry of Foreign Affairs did not provide permits for them to enter and work in Lebanon, for example UNHCR apply for 20 permits but they receive only 5, they request 20 cars and receive 5, there was major restrictions for this agency’ PM-12.*

A few of our participants indicated that they never expected that the crisis would last that long and would displace such a high number of refugees, hence they were providing short term solutions for the crisis:

*‘It was an emergency response to an event that happened suddenly, and it wasn’t clear for how long it will last’ PM-10*

*‘Yes, short-term, we were living as if Syrians will return to Syria the next day’ PM-12*

According to one participant, one of the reasons for the poor response is ineptitude. There was lack of expertise in dealing with a crisis of such magnitude. The respondent specifically stated: *‘I really don’t know whether we have the know-how and the level of knowledge and skills at the ministry that allows us to conduct such a response for refugees’ PM-06.*

### Policy stream: How health integration policy entered the political agenda

Per Kingdon’s Model, ideas for solutions start to float, some prosper leading to a policy, and others fade. Advocates promoting specific policy or program options, defined by Kingdon as the “policy entrepreneurs,” must be cognizant of policy windows and act quickly before the opportunity passes [32].

As more refugees crossed the border, health conditions and the fear of spilling over to the host community presented a window of opportunity to start discussions for an integrated health policy mediated by international humanitarian agencies such as UNHCR and WHO. The Lebanese Response Crisis Plan (LRCP) was the roadmap for the integrated health policy.

#### INGO creating a fragmented parallel system

The lack of clear government policy led humanitarian and UN agencies and governmental bodies to start providing, subsidizing, and financing health services for Syrian refugees. These services were poorly coordinated [42]. The UNHCR and other international actors created their own health delivery and financing channels that generated a parallel system and fragmented the health response [21]. This process was outlined by one participant discussing subsidy to hospital services by UNHCR: *‘As public hospitals, we didn’t have an international funding in exchange of providing health services to Syrian refugees. In that regard, the UNHCR reimbursed us for the Syrian refugees’ medical tariffs’ PM-02.*

#### The timid beginning for policies started with the polio outbreak in 2013

In 2013, a polio outbreak started among Syrian refugees. The potential for infection spread in refugee camps caused great concern among refugees themselves, host communities, and local authorities, since they presented a suitable environment for massive viral dissemination concern [54, 55]. The MoPH issued an immediate campaign response to start vaccinating all children residing in Lebanon below 5 years [56]. This was perhaps the first official response in that crisis: *‘This is an important policy that was put in place by the MoPH to avoid any outbreak in the country. This policy was successful because since the beginning of the Syrian crisis we haven’t had any further polio cases in Lebanon (0 case)’ PM-05.* In addition, the MoPH in collaboration with UNHCR and the General Security Forces set up vaccination centers at the borders to vaccinate Syrian children [57].

#### Lebanon crisis response plan (LCRP) 2015–2017

From those timid beginnings, UNHCR was able to add the integration policy into the political agenda after several attempts to engage the local authorities to act as the main international organization concerned with refugee health. This came into effect in late 2014 and early 2015, three years into the crisis. In December 2014, the government issued the LCRP, which iterated the strategy for integrating refugees into the health care system and simultaneously assisting the host community. This was a two-year national plan (2015–2016) in which the MoPH developed a Health Response Strategy [58, 59]. These plans and strategies aimed to detail and coordinate all funding sources and activities performed among local and international partners to cater for the needs of displaced Syrians and the poorest Lebanese; to strengthen the capacity of national and local service delivery systems to improve access to and quality of basic public services; and to reinforce the economic, social, environmental, and institutional stability. They aimed to shift the response from the initial humanitarian response that targeted the most urgent and life-saving health needs of the displaced population to strengthening the health system and increasing its resilience capacity: *‘From the start of the refugees’ influx crisis, a yearly plan was elaborated in collaboration with UNHCR to respond to the Syrian refugees needs in addition to the host communities in Lebanon. This was done in the LCRP framework which is the main policy [plan] used to address the Syrian refugee crisis’ PM-09.*

This inclusive form of governance, based on participation, transparency, and accountability, was essential in addressing all the building blocks of a health care system. It provided guidance on leadership and governance, service delivery, health system financing, health workforce, supplies, and health information systems. LCRP was meant to organize a successful emergency response, build resilience of the health system, and develop a successful surveillance system [21]. The LCRP plan in coordination with the UNHCR was multiphasic: 1-coordinating with the national authorities the offerings of health care services to refugees while supporting the host communities; 2- assuring implementation of the plan; and 3- while simultaneously financing those services.

1- Coordinating with the national authorities for a health integration policy.

UNHCR worked with MoPH to coordinate the process through which the health integration will occur, addressing key players at the leadership and governance level. As one policymaker related: *‘The ministries, on top of them the MoPH, were involved in the negotiation and coordination of the response to the refugees’ crisis’ PM-10.*

It became obvious that relying on the existing infrastructure would be the best avenue to support integration. This meant using the premises of health care institutions as infrastructure to provide refugees with health care services: *‘The primary source relied highly on existing national resources, so when the UN or INGOs came they supported in part but they relied on the existing national health system be it in the infrastructure or in the electricity, or water…. PM-07.*

Then, international organizations coordinated the service delivery they would support in order to meet the needs of refugees. They negotiated existing services, e.g., chronic disease management, enhanced the information systems to capture both refugee and host communities, and added tools for integrating refugees with different departments at MoPH. They looked into the primary, secondary, and tertiary levels of care and the mechanisms for integrating the refugees into the existing systems.

In the beginning, the UNHCR selected certain primary health care centers based on the concentration of refugees in their communities but eventually this approach has changed overtime.

*‘Their choice of which PHC [primary healthcare center] to go to was determined by the location of the PHC. Definitely, this changed with time especially with the allocation of a huge amount of funding to the Syrian refugee crisis. Of course, with time the response to the Syrian refugee crisis became more organized especially with the elaboration of the Lebanese Crisis Response Pan (LCRP)’ PM-10.*

2- Assuring implementation of the plan.

To assure the implementation of the LCRP activities, a team that initially was made up of UNHCR and MoPH, later joined by WHO, were responsible for ensuring the implementation is occurring as planned: *‘Alongside UNHCR, there was a teamwork that was executing all the program on the ground. This execution was done through Development Services Centers. I named UNHCR since it was the main organization concerned directly in the refugees’ crisis’ PM-09.* Hence, the Government of Lebanon represented by the MoPH started to lead the health integration policy: *‘As for the MoPH, they were either the lead or the co-lead of those policies and being the co-lead of the health sector’. PM-01.*

3- Financial support.

The financial support strengthened the health care system to withstand the pressure. First, it was essential for covering the fee for services.

*‘the financial support has been done through UNHCR and the international donors…they have been covering all the weaknesses and the gaps in the Lebanese government and the provision of services’ PM-04.*

Second, the financial support supported centers with human resources, equipment and programs, and training staff. More personnel were hired such as nurses, nutritionists etc.… More equipment and medications were provided to the centers. Programs such as mental health, gender-based violence prevention, and malnutrition programs were introduced. Training staff on the delivery of those programs were also provided through this financial support.

*‘UNHCR as agency or from other agencies like UNICEF sometimes, IMC, UME, Relief International and other NGOs. The support of the centers is in the form of health systems strengthening. So, we are strengthening this system in order for it to be able to withstand the pressure caused by the increased demand and the scarcity of resources. How? Through supporting these centers with human resources, equipment, subsidization of consultation, medication, nutrition services, mental health services, sexual and intellectual health services. This support is either in the form of commodities, or trainings and capacity buildings, or vaccination services. This way the sector supported the integration of refugees in the primary healthcare system’ PM-11.*

#### Shape of the health integration policy in Lebanon

##### Primary health care services

The MoPH primary health care (PHC) network was a key stone in the health system response to refugees. Initially, through its PHC network of PHCCs and dispensaries run by NGOs, the MoPH provided PHC services to the vulnerable and poor Lebanese and presented an affordable alternative to the private costly ambulatory care [49].

In 2014, in order to revamp the mental health system and expand services, the Lebanese MoPH in conjunction with WHO, UNICEF, and the International Medical Corps introduced the first National Mental Health Programme that provided mental health services for those who cannot afford the private sector including refugees.

Starting with the primary health care services, three consecutive policies were issued to integrate Syrian refugees into the primary health care services:

*‘This is an important policy that was put in place by the MoPH to avoid any outbreak in the country. This policy was successful because since the beginning of the Syrian crisis we haven’t had any polio cases in Lebanon (0 case). The second policy, was allowing the Syrian refugees to access chronic medications through the national health system. As you may know, the YMCA alongside the MoPH and with their donors they are providing chronic medications to beneficiaries including Syrian refugees. This inclusion of Syrian refugees in the chronic medication provision system was a great achievement. The third policy, was the inclusion of the Syrian refugees in the Primary Healthcare system that were able to access services at PHCs. I think these are the 3 main high-level strategies that allowed the refugees to access health services in Lebanon’ PM-05.*

And according to another participant, including the Syrian refugees in the chronic medication program was the first step towards integration: *‘When the MoPH started to integrate Syrian refugees in the YMCA cohort, this constituted the start of the Lebanese MoPH’s response to the refugee crisis. As this allowed Syrian refugees to take chronic medications systematically from PHCs. Before that inclusion in the YMCA, Syrian refugees would benefit from chronic medications free of charge from [PHC clinics affiliated with international organizations] and PHCs that have an NCD program’ PM-10.*

There was a consensus that integration at the primary care level was successful, depending on whether the primary health care center is supported by UNHCR or not. As one policymaker stated: *‘According to The Vulnerability Assessment of Syrian Refugees in Lebanon (VASyR), 90% of the Syrian refugees who needed care were able to access it in 2021’ PM-11.* Other policymakers also mentioned *‘Actually, all the PHCs accredited by the ministry of public health are in theory and in practice accessible to Syrian refugees although …. you would always find some discriminatory practices.’ PM-01.*

The provision of the health care to Syrian refugees was impeded by the weaknesses of the health information system that restricted the ability of the relevant authorities to determine the health needs of the Syrian refugees. However, starting 2016, some initiatives to develop national health information systems took place. In particular, the Health Information System (HIS) at the MoPH to link and unify the network of PHC centers referred to as PHENICs was developed [60]. This HIS registered all beneficiaries of the services including refugees, the services they receive and the outcomes of care. Thus, it is used to monitor and report on key performance indicators of the PHC services.

##### The secondary and tertiary levels of care

The secondary and tertiary levels of care were more challenging to integrate. Healthcare facilities were strained by scarcity of financial resources that hindered their capacity to admit patients and cover hospitalization costs. The health integration policy had to select which services to cover partially with co-payment from the beneficiaries, which health care institutions to deliver those services, the eligibility criteria for coverage, and co-payment. Displaced Syrians found it very challenging to cover their cost shares of hospital bills [61]. In a nutshell, the secondary and tertiary services were patchy: *‘It was harder to integrate Syrian refugees in the hospital care system because of the capacity issue. If they are integrated in the hospital care system, they will be admitted at the expense of the MoPH that will be covering their hospitalization cost. Also, there is no network of private and public hospitals that are linked to the MoPH. There are only public hospitals that are linked to the MoPH, and these public hospitals don’t have the finances or resources to receive all the Syrian refugees’ PM-11.*

First, in terms of services, not all services were covered and even if covered, the co-payment posed a challenge for the refugees: *‘This has been particularly difficult for individuals with chronic health issues and those that require very costly and specialized surgeries, so finding the appropriate source of funding in these cases has been really difficult’ PM-03.*

Second, the hospitals that provide services for refugees were mainly public or private hospitals in the country’s periphery:

*‘Their integration is simply due to the fact that they had to be offered secondary health services either by private or public hospitals. Private hospitals did not want to receive the Syrian refugees because this meant they will be reimbursed the MoPH’s tariffs. So, the peripheral private hospitals and public hospitals took the initiative and received these refugees’ PM-02.*

Third, unregistered refugees were at a further disadvantage since they were not covered by UNHCR: *‘At the hospital level UNHCR cover only registered refugees, the unregistered are paying out-of-pocket. In other words, you are not denying them services at the hospital level, you are not denying them the provision, and you are denying them the financing’ PM-0.*

### Political stream

Last, the political stream refers to the lobbying of key interests’ groups to gain wider acceptance of the policy [32]. The political streams that supported the integrated policy included the unofficial implementation of a ‘no camp policy’ [62], the previous Palestinian refugee experience in Lebanon, policy think tank, additional NGO actors, and certain communities supporting the refugees.

#### The Palestinian refugee issue and the ‘No camp policy’

The governments of Lebanon consider fleeing populations as “displaced,” administratively labeling them “guests” rather than “refugees.” Therefore, the Lebanese government informally adopted a non-encampment policy [44, 61]. Consequently, Syrian refugees lived within host communities, in informal settlements, and were scattered across around 1,800 locations in 2015 [62]. This policy can be linked to several threads within Lebanese domestic politics. In some sectors of the Lebanese population and political class, there exists a fear that primarily Sunni Muslim refugees, if they were allowed to settle permanently in Lebanon, would cause a sectarian imbalance given the Lebanese confessional system, which allocates government positions and parliamentary seats proportionally based on sect [19, 44]. As one interviewee articulated this sentiment: *‘I am not with the idea of integration, because integration could hint at other meanings, such as permanent domiciliation, I prefer to use the term absorbing, absorb the Syrian refugees’ PM-12.*

This stance can be situated in the historical Lebanese experience with Palestinian refugees, who have existed in Lebanon since 1948. Approximately 50% of Palestinian refugees live in camps administered by the United Nations Relief and Works Agency for Palestine Refugees in the Near East (UNRWA), which manages education and primary healthcare services. Palestinians access Palestinian Red Crescent Society (PRCS) hospitals for some secondary care and Lebanese public system for much of their secondary and tertiary care [63]. The camps, which are Palestinian governed under the terms of the 1969 Cairo Agreement, historically provided spaces for Palestinians’ social and political mobilization in the late twentieth century, culminating in extensive Palestinian involvement in the 1975–1990 Lebanese civil war. The 12 contemporary camps are currently characterized by extremely high levels of poverty and social exclusion in addition to being seen by some Lebanese as dangerous locations and hotbeds for radicalization. The different facets of the Palestinian issue in Lebanon—from social exclusion to armed mobilization (including against Syrian authorities as well as the Lebanese state) shape Lebanese political factions’ interpretation of the Syrian refugee crisis, leading many to declare the establishment of refugee camps as an unacceptable policy [17]. As one interviewee noted: “*So, this country didn’t know which policy to follow and all what they knew is that they didn’t want something permanent. So, they tried not to make camps like in the case of the Palestinians” (PM-04).* Another interviewee emphasized: ‘*Since the beginning, the government adopted a “no camp policy”. We can’t say if it was a good or bad decision but for the government, the creation of camps could lead this crisis to become protractive and a long-life crisis like the Palestinian refugees’ situation’ (PM-10).*

Consequently, Syrian refugees found accommodation within or adjacent to host communities, whether in informal tented settlements, rented apartments, Palestinian refugee camps, or makeshift accommodations in abandoned buildings or mosques. The UNHCR identified around 1,800 locations that housed Syrian refugees in 2015 [64]. This was also affected by Lebanon’s inability to control its borders with Syria and the illegal border crossings, influenced by a lack of political will and divisions among political parties [65]. The non-encampment policy prevented the creation of a parallel humanitarian health system as with the Palestinians and facilitated the process of the health integration policy, albeit delays, economic shortfalls, and political tensions.

#### Additional INGOs actors

The key actors of the integration health policy were UNHCR, MoPH, and WHO; other actors joined efforts to fund and provide comprehensive services such as the United Nations Office for the Coordination of Humanitarian Affairs (UNOCHA) and the World Bank. On the other hand, some NGOs, such as Médecins Sans Frontières (MSF), were also providing health care, either through their own established health facilities or by subsidizing health care provided by the existing Lebanese public health facilities.

*‘Later on, other actors came into play such as OCHA, the ministries and other UN agencies. The coordination with UNHCR was in the form of meetings that consisted of brain storming and dividing the work to avoid the duplication of tasks as well as developing the 3 W and 4 W matrix. This coordination with UNHCR regarding the Syrian refugee crisis lasted for years’ PM-10.*

At times funders were earmarking the funds for specific refugee population and host communities.

*‘Some of the donors actually request that a certain amount of the budget is allocated to targeting refugees. For example, the last project with the World Bank, the World Bank allocated a specific amount, I think it was 40% to target Syrian refugees living in Informal Tented Settlements (ITSs) and 60% to target the host community and other non- Syrian migrants such as the Palestinians and domestic workers’ PM-06.*

#### Policy think-tank organizations advocating on behalf of the refugees

Certain think tank policy organizations took the lead in advocating for the rights of refugees, thus acting as lobbyist and activist to support the policy. One example is the efforts to improve birth registration of newborns and to raise awareness about the implications of poor registration. In principle, this advocacy should be translated into action to increase accessibility to services.

*‘The institute being an academic think tank we take it a step further similar to K2P and take it towards policy. So, for example, at some point, there was the issue of birth registration of newborns mainly among Syrian refugees and Palestinian Refugees from Syria. So, we had a series of meetings where we included UNRWA, UNHCR, NRC, and the ministry of interior. In that case we took the data from NRC, and we didn’t produce it ourselves, so we don’t have duplication. In these meetings, we didn’t solve the problem but we were able to show the current state and explain the implications’ PM-04.*

#### The community played a role in ensuring the implementation of health integration policy

The initial response to the Syrian influx at the social level was a supportive and sympathetic one from a humanitarian lens [53]. Hence, the host community was sympathetic to the plight of the Syrian refugees and offered assistance.

*‘At the beginning of the crisis, there was a lot of sympathy in Lebanon towards the Syrian refugees which translated into having several NGOs dealing with them’ PM-12*

Certain host communities shared the same religious or political beliefs as the refugees; hence religion and politics formed the basis for better integration, health and otherwise.

*‘Second, there was a huge Islamic and Arab support to the Syrian refugees' crisis, taking into account that it was a supported cause, thus the local community borne the burden instead of the government in addition to local NGOs (some were religious organizations and others were civil organizations) and they played the largest role’ PM-12.*

### Overview of the financial mechanism and reporting

Multiple national and international organizations and governmental agencies were involved in the provision and financing of health services to Syrian refugees. UNHCR was the entity that managed the funds from foreign donors, which were then distributed through a variety of national and international NGOs. Noting that the MoPH did not directly receive any of this funding; instead, it worked with foreign organizations to ensure that the funds were distributed through various national and international NGOs to the relevant priority areas and populations [21]. Another indirect source of funding was through the Government of Lebanon: *‘Also, donations from the MoPH when it comes to vaccines and medications’ PM-05.*

For Primary Care, the Syrian refugees benefited from subsidized PHC services in PHCCs and dispensaries supported and funded by the UNHCR or its international partners. These PHCCs were mostly run by NGOs and are part of the MoPH’s national PHC network [60]. In 2016, the MoPH developed the Emergency Primary Healthcare Restoration Project in collaboration with the World Bank to deliver a package of free PHC services (Essential Benefits Package) to underprivileged Lebanese, identified by the National Poverty Targeting Program of the Ministry of Social Affairs, as well as to refugees [66].

*‘Now, the second contribution is indeed the donor contribution, the partner contribution and there is as well the French, the German, and so on. In that regard, I think the first donor in the health system is the European Union through the Madad Trust Fund’ PM-01.*

*‘The primary source of financial support for the refugees’ health services is the iNGOS whether it is ECO, the embassies, USAID, and MSF that has its own fund’ PM-10*

Sustainability of financing this model was an important concern for many: *‘The system is thus relying on funding that is not sustainable, how is it supposed to last and endure? The sustainability of the production of indicators and statistics is challenged, and one of the major reasons is financing. Once the funding stops everything stops, the funding sometimes is also related to the political climate in the country’. PM-08.*

## Discussion

This study discusses the factors that influenced the emergence of the policy on integrating refugees in the Lebanese health system on the government agenda. It demonstrates how this policy was put forward to address the urgent health needs of sheer number of Syrian refugees supported by global actors and shaped by political and sectarian dissensions of the refugee crisis. The integration was facilitated and coordinated with the government, which was politically divided over the refugee situation, but eventually had to acknowledge the fact that refugees were on Lebanese territory and needed health care services. Integrating refugees into the existing health care system was the most viable solution. However, integration occurred mainly at the PHC level, whereas the secondary and tertiary health care level were based in mainly public hospitals and covering essential services with co-payment. Another key finding in this study is that in fragile countries such as Lebanon, global actors such as UNHCR and WHO among others are the main entrepreneurs in integrating refugees into health care systems. Other important players were NGOs on the ground, certain host communities who welcomed and supported refugees, and think tank organizations who advocated for better services for refugees. It is also important to note that the previous experience with the Palestinian refugee crisis and the fear of domiciliation were important political factors that pushed policymakers towards integrating refugees in the Lebanese system as opposed to establishing official camps and parallel healthcare systems.

Our findings are consistent with studies on integrating refugees in host community health care systems in low, middle income, and high-income counties. In low- and middle-income refugee-hosting countries such as Jordan and Uganda, the healthcare system was already burdened by scarce resources, poor infrastructure, and a high prevalence of diseases, which made it difficult to provide the host community with universal and equitable healthcare services [10, 67]. The influx of refugees placed an immense strain on already fragile heath systems, which compromised the availability of healthcare services for refugees and hindered their integration into the existing health system. These dynamics have impeded access to vital healthcare resources and further exacerbated health disparities [10, 67]. Hence, integrating refugees in LMICs countries can be challenging due to their fragile healthcare systems. High-income countries face different types of obstacles, including limited healthcare access for undocumented migrants and pending legislative reforms for equal access [68, 69]. Moreover, cultural barriers, resource allocation, and societal attitudes hinder refugee integration. For instance, only 13 EU member states offer free interpreting services, and there is currently no official health strategy targeting migrants [69].

Undoubtedly, the Lebanese integration policy facilitated financial accessibility and availability of services for the vulnerable Syrian refugee population while providing a thrust to strengthen the host community health care system and specifically primary health care services. Even before the Syrian refugee crisis, Lebanon endured repeated wars, economic shocks, and political instability, all of which weakened its health care system [50] and made essential health services less available to Lebanese. For example, mental health disorders in Lebanon are common, with an estimated 1 in 4 Lebanese residents having had one or more mental health disorder in their lifetime. However, essential mental health services remain limited through the public health care system. Lebanese instead rely on the out-of-pocket private health care system to attend to the mental health care, hence accessible to those who can afford them and essential health services were not readily available for the Lebanese [70]. After the onset of the refugee crisis, mental health services and other much needed services, equipment. and capacity building activities, benefitting both Lebanese and Syrians, were introduced into the public health care system, hence increasing accessibility and availability through the public health care system [71].

This paper does not specifically address the integration of gender-based services. However, given the majority of all Syrians registered by UNHCR are women and children, with more than half (54%) of the refugee population being under the age of 18, it is worth pointing that although the integration policy included sexual and reproductive health coverage and children’s programs, the quality of those services was poor due to inequitable access to services [46, 72]. To improve the quality of services, Fouad et al. suggested increasing funding for training staff on family planning services, acquiring more equipment, and more coverage for different sexual and reproductive health services [73].

The negative impact of the health integration policy is also tangible. First, the massive number of refugees residing in Lebanon caused a severe stress on the health care system. Providers’ workload increased exponentially, specifically in primary health care centers. In the beginning, health care providers were more compassionate and giving, gradually, however, they started reporting compassion fatigue. Workload affected providers’ physical and psychological welfare while value of the additional financial incentives they received for serving refugees started to fade. Additionally, most refugees using the integrated and subsidized serves have poor health literacy. Their precarious living conditions affect their health outcomes, hence their disease profiles caused additional challenges to the health care providers [74].

Further, increased demand for health care has negatively affected the quality of service provided for both refugees and local populations due to overcrowding, increased waiting times, and decreased provider job satisfaction due to fatigue, burn out, and increased workload at the health care professionals’ levels [22, 42, 75]. The lack of trained staff to respond to new encountered cases (such as leishmaniosis, scabies) also caused poor quality and responsiveness to refugees’ needs [75].

Several studies reported on the challenges that both Syrian refugees and Lebanese face accessing public health services. Out-of-pocket payments, security concerns, and transportation were among the barriers accessing essential services such as antenatal care and deliveries [14]. Some refugees rationed their utilization of health care services due to those constraints [29]. Lebanese citizens, on the other hand, had different challenges with the integration of services. Crowdedness and long wait times in public health services used by Syrian refugees made many Lebanese avoid those services. Also, at the outset of the policy, the difference in payment between Lebanese and Syrians, where the former had to pay equivalent of $10 for a consultation while the latter had to pay $2–3, were sources of frustration for the Lebanese community [67].

In this study, we identified a gap in the continuity of care between the primary health care centers and hospitals. Individuals can easily access, both geographically and financially, primary health care services but when they were asked to use hospital services, then accessibility was limited due to out-of-pocket payment and the limited range of services covered. Mc Murray et al. in Canada, proposed establishing a designated relationship management office supported by internationally trained graduates sharing the same culture as service users. Their role was to support the navigation between public and private [76]. This approach increased access to a primary care provider on one hand and reduced wait times. More importantly, using culturally competent health care providers at these centers would mitigate unnecessary services. In the context of Lebanon, the relational, informational and management continuity of care can be improved using a relational officer to reduce the accessibility barrier.

Applying Kingdon’s framework in the Lebanese context has multiple strengths. First, it allowed the understanding of how multiple factors at the problem, policy and political levels came together to shape the integration of refugees in the Lebanese health system. In specific, it highlights’ key stakeholders’ perceptions, including those of ministers of health, to reflect how high-level policymaking occurred, unpack the policy formation process and elucidate the forces that helped formulating a policy. Additionally, the multi-method qualitative approach the framework facilitated allowed us to triangulate different forms of data, minimizing the effects of recall bias and providing robust results.

Despite the strengths of the Kingdon’s framework, critics indicate several shortcomings to its use, two important ones apply specifically to Lebanon. First, there may be other critical factors that may have not been fully captured by any of the streams [76]. These factors may have been random yet influenced the process. For example, it is possible that in certain communities, the tension between host and refugees may have precipitated politicians to act in order to avoid wider xenophobic reactions in the country leading to unrest [77]. Second, personal interests [78, 79], particularly those of politicians interested in sustaining their elections by their constituents or those that are directly or indirectly benefiting from the funding for the refugees, may have played a role in the process, yet not captured in the forces. A third limitation is its reliance on primarily qualitative study designs and the limited ability to use quantitative measures to test the hypotheses associating between these streams and the policy [80, 81].

Although Lebanese health system showed resilience during the Syrian refugee crisis, our analysis showed that political factors and global actors played a pivotal role in the emergence of the policy for integrating of Syrian refugees into Lebanon's healthcare system [21]. Reforming the health sector and creating mechanisms to ensure universal health coverage such as sustainable financing modalities and improving the primary care system are essential to prepare the country to respond to any emergency and crisis, rather than relying on “quick-fix” approach of policymakers. Finally, further research is needed in order to examine the implications of the integrated approach on the Lebanese health care system, health care providers and beneficiaries, both Syrian and Lebanese.

## Conclusion

This study highlights the role of global actors, namely UNHCR and WHO, as the main entrepreneurs in integrating refugees into the Lebanese health care system. They coordinated with the government, which was itself divided over the refugee situation. Given the magnitude of the crisis, refugees’ immediate health needs, and Lebanon’s political history, integrating Syrians into the existing health care system emerged the most viable solution. The integration was mainly at the primary health care level, whereas the secondary and tertiary health care level were based in mainly public hospitals and covering essential services with co-payment.

The integrated policy has been established for over a decade. The mutual benefits to both host and refugee communities are tangible. However, there remain many challenges that threaten the integrated system. Primarily, massive stress on the health care system, especially in the context of economic collapse, has jeopardized the quality of services, the well-being of health care workers, and the financial sustainability of the model. These findings underscore the ad-hoc non-systematic approach with which the policies around refugee health response were made and the influence of political factors. This calls the government of Lebanon to develop a comprehensive, multidisciplinary emergency preparedness plan. This also calls for a universal health coverage law in Lebanon that provide universal access to high quality health services for all Lebanese and residents. This study also provides insights to global actors to design sustainable models for integrating refugees in fragile health systems.

## Supplementary Information

Additional file 1.

## Data Availability

Data is available upon reasonable request from the corresponding author.

## Abbreviations

UNHCR: United Nations High Commissioner for Refugees
WHO: World Health Organization
INGOs: International non-governmental organizations
PHC: Primary health care
MOPH: Ministry of Public Health
UNRWA: United Nations Relief and Works Agency for Palestine Refugees in the Near East
LMICs: Low and middle-income countries
PRCS: Palestinian Red Crescent Society
UNICEF: United Nations Children’s Fund
