# Supplementary material for: Exploring factors that shaped Syrian refugees integration into Lebanon’s national health system using Kingdon’s Multiple Streams Framework
Source: Confl Health. 2025 Jul 10;18(Suppl 1):79. doi: 10.1186/s13031-025-00680-2 (PMC12243152; doi:10.1186/s13031-025-00680-2)
Supplement: Supplementary file 1 — Additional file 1. [file 13031_2025_680_MOESM1_ESM.docx]

**Additional file 1**

**Electronic database search**

Database: **Ovid MEDLINE(R**) and Epub Ahead of Print, In-Process & Other Non-Indexed Citations and Daily <1946 to September 12, 2019>

Search Strategy:

--------------------------------------------------------------------------------

1 refugee*.ti,ab. (9544)

2 Refugees/ (9523)

3 displaced.ti,ab. (33948)

4 Lebanon/ (3953)

5 Lebanon.ti,ab. (4067)

6 1 or 2 or 3 (45960)

7 4 or 5 (5584)

8 6 and 7 (314)

***************************

**PubMed Search strategy (September 16, 2019)**

"refugees"[MeSH Terms] OR "refugees"[All Fields] OR "refugee"[All Fields]

"Lebanon"[MeSH Terms] OR "Lebanon"[All Fields]

#1 AND #2 (366 hits)

**Scopus Search strategy (February 13, 2020)**

( TITLE-ABS-KEY ( *Lebanon* ) )  AND  ( ( TITLE-ABS-KEY ( *refugee** ) )  OR  ( TITLE-ABS-KEY ( *displaced* ) ) )  (554 hits)

**Additional file 2**

**List of agencies and organizations searched**

**Government agencies/organizations**

| Government organization | Link to website | Date searched | Number of hits retrieved | Number of documents we might include |
| --- | --- | --- | --- | --- |
| Council of ministers | <http://www.pcm.gov.lb/> | 17/12/2019 | 5 | 1 |
| Ministry of Labor | <https://www.labor.gov.lb/> | 17/12/2019 | 54 | 27 |
| Ministry of Public Health | <https://www.moph.gov.lb/> | 17/12/2019 | 61 | 29 |
| Ministry of Social Affairs | <http://www.socialaffairs.gov.lb/> | 17/12/2019 | 62 | 10 |
| Ministry of interior/ General security | <http://www.interior.gov.lb/> | 17/12/2019 | 74 | 12 |
| Ministry of economy | <https://www.economy.gov.lb/> | 17/12/2019 | 50 | 2 |
| Ministry of education | <https://www.mehe.gov.lb/> | 17/12/2019 | 63 | 11 |

**International agencies/organizations**

| International agency | Link to website | Date searched | Number of hits retrieved | Number of documents we might include |
| --- | --- | --- | --- | --- |
| Danish Refugee Council | <https://drc.ngo/> | 27/11/2019 | 7 | 2 |
| Human Rights Watch | <https://www.hrw.org/> | 27/11/2019 | 296 | 12 |
| ILO | [https://www.ilo.org](https://www.ilo.org/) | 10/12/2019 | 5 | 2 |
| IMC | <https://internationalmedicalcorps.org/> | 10/12/2019 | 78 | 25 |
| IOM | <https://www.iom.int/> | 10/12/2019 | 130 | 23 |
| International Rescue Committee – IRC | <https://www.rescue.org/> | 11/12/2019 | 298 | 16 |
| MDM | <https://www.medecinsdumonde.org/en> | 11/12/2019 | 4 | 0 |
| Mercy Corps | <https://www.mercycorps.org/> | 11/12/2019 | 87 | 11 |
| MSF | <https://www.msf.org/> | 11/12/2019 | 80 | 7 |
| Care | <https://www.care.org/> | 11/12/2019 | 59 | 7 |
| Syria Public Health Network | <http://www.syriahealthnetwork.org/> | 11/12/2019 | 1 | 0 |
| Norwegian Refugee Council | <https://www.nrc.no/> | 11/12/2019 | 80 | 15 |
| Save the Children | <https://www.savethechildren.net/> | 11/12/2019 | 14 | 4 |
| UN DESA | <https://www.un.org/development/desa/en/> | 11/12/2019 | 1 | 0 |
| UNDP | <https://www.undp.org/> | 11/12/2019 | 98 | 18 |
| UN ESCWA | <https://www.unescwa.org/> | 11/12/2019 | 40 | 5 |
| UNFPA | <https://www.unfpa.org/> | 12/12/2019 | 152 | 16 |
| UNHCR | <https://www.unhcr.org/> | 12/12/2019 | 276 | 36 |
| UNICEF | [https://www.unicef.org](https://www.unicef.org/) | 12/12/2019 | 279 | 44 |
| UN Lebanon | <http://www.un.org.lb/> | 17/12/2019 | 42 | 10 |
| UN OCHA  (relief web) | <https://www.unocha.org/> | 26/11/2019 | 74 | 44 |
| UNRWA | <https://www.unrwa.org/> | 12/12/2019 | 166 | 17 |
| WFP | <https://www.wfp.org/> | 12/12/2019 | 56 | 0 |
| WHO | <https://www.who.int/> | 12/12/2019 | 223 | 21 |
| WHO EMRO | <http://www.emro.who.int/index.html> | 12/12/2019 | 24 | 7 |
| World Bank | <https://www.worldbank.org/> | 16/12/2019 | 109 | 2 |
| Amnesty International | <https://www.amnesty.org/en/> | 9/1/2020 | 9 | 1 |

**National and regional organizations and research institutions**

| National/ regional agency | Link to website | Date searched | Number of hits retrieved | Number of documents we might include |
| --- | --- | --- | --- | --- |
| ABAAD | <https://www.abaadmena.org/> | 16/12/2019 | 38 | 8 |
| IDRAAC | [http://www.idraac.org](http://www.idraac.org/) | 16/12/2019 | 12 | 0 |
| Caritas | [https://www.caritas.org](https://www.caritas.org/) | 16/12/2019 | 49 | 1 |
| Amel association | <https://amel.org/> | 16/12/2019 | 43 | 0 |
| IFI - refugee program | <https://www.aub.edu.lb/ifi> | 16/12/2019 | 42 | 12 |
| GHI - refugee program | <https://ghi.aub.edu.lb/> | 16/12/2019 | 17 | 2 |
| Makhzoumi Foundation | <https://makhzoumi-foundation.org/> | 16/12/2019 | 8 | 6 |
| Basmeh & Zeitooneh | <https://www.basmeh-zeitooneh.org/> | 16/12/2019 | 1 | 1 |

**Additional file 3**

**Interview email invitation**

I hope this email finds you well.

We are pleased to invite you to participate in a research study on the integration of refugees into national health systems and the response of the Lebanese health system to the refugees’ crisis. The study is conducted by the Faculty of Health Sciences at the American University of Beirut in collaboration with the Johns Hopkins University.

The purpose of this study is to inform public policy and decision-making at the national, regional and global levels concerning the integration of refugees into national health systems, and ultimately enhance equity and strengthen sustainable health services for all.

We are asking you for an interview (online or face to face based on your preference) that will last between 30 and 45 minutes. We will be conducting interviews with policymakers (from the health sector, refugee affairs, and other relevant government departments) and international actors working with refugees.

Your name will remain confidential, and the information provided by you will be anonymous. You will be provided a copy of the final publication.

Please let us know your willingness to participate and if yes, your availability so we can schedule the interview.

Please do not hesitate to contact us for any further information.

Your participation is highly appreciated.

Best Regards,

**Additional file 4**

**Policymaker/stakeholder interview questions (English Version)**

1. Could you briefly describe your main role and responsibilities vis-à-vis refugees in Lebanon, given your position as XX (minister/ general director, etc.)?
   1. To what extent do you have direct contact with refugees (e.g., visiting hospitals and/or informal tented settlements)?
2. Given the current health situation in the country, I would like to start with some questions about refugees and COVID-19, before we shift the discussion to the pre-pandemic era.
   1. To what extent has your office organized itself to conduct a COVID refugee response?
   2. What are the key developments in refugee-related policies since the start of the COVID-19 pandemic?
   3. Which organizations would you say are the major players in Covid-19 refugee response?
   4. How do you coordinate with them?
   5. What are the major funding sources (e.g., donors, OOP etc.) for COVID-19 response in refugee communities (for example, for prevention, detection, isolation, and control measures, testing and treatment)?
   6. Are COVID-19 testing and treatment easily accessible for refugees?
   7. Are testing and treatment free to all refugees, covered all or in part through financing agents, or full cost to all?
   8. Does refugees’ access to testing vary regionally?
   9. How do you think the care received by Syrian refugees in Lebanon compares to the care local populations receive for COVID-19 (including vaccine deployment)?
   10. Is there anything else you would like to touch upon related to the refugee response during the COVID-19 crisis or the current situation before we move forward to the pre-pandemic era?
3. Let us go back to the pre-COVID period. In your opinion, what were the key health policy developments since the Syrian refugees began arriving here in 2011?
   1. Probe: [questions regarding country-specific policies based on desk research]
   2. What is your opinion on these policy changes?
   3. What do you think prompted these developments?
   4. Who or which organizations do you believe had central roles in these policy changes?
   5. How would you characterize the role of the MoH in these policy shifts?
   6. How do you believe these policy developments affected health services provision?
   7. How do you believe these policy developments have affected front line healthcare providers?
   8. In a few sentences, could you describe, in your opinion, what the most important aspects of [country’s] prior experience with healthcare for refugees are for the current situation?
4. What would you name as the primary sources of financial support for refugee health services in Lebanon?
   1. In your view, how effective have these funding systems been?
   2. How has the refugee situation affected government spending on health care?
   3. How do you think donor funding has affected the quality of healthcare facilities can provide:
      1. To refugees?
      2. To locals?
   4. In your view, what are/have been the main challenges in managing healthcare funding? (probe: corruption?)
   5. Did these funding sources change or shift after the COVID-19?
5. In general, how do you think the healthcare received by Syrian refugees in Lebanon compares to the healthcare local populations receive?
   1. What changes have you observed in health service delivery for refugees and host populations over time?
   2. What kinds of economic and financial factors do you see as affecting care for each population?
   3. In your professional capacity, what kinds of regional differences, if any, are you aware of when it comes to refugees’ versus local populations’ experience of health care?
   4. What would you say are there local-level conditions or relationships most likely to affect refugees’ healthcare experience?
6. On a scale of zero to ten, with zero being “not integrated at all” and ten being “completely integrated,” to what extent do you think the refugees are integrated into the health system in Lebanon?
   1. Could you explain your answer in a few sentences?
   2. What does it mean for refugees to be “integrated” into the Lebanese healthcare system?
7. If you were at an international conference with someone who has the exact same job as you in [Colombia/Bangladesh/other refugee receiving state]. What advice would you give them, given your experience?
8. Is there anything I should have asked you but did not? Would you like to add anything else?

*Thank you very much for your time; we appreciate your participation in this study.*

**Additional file 5**

**The thematic framework by (Crabtree & Miller, 1992).**

1. Data familiarization

At first, the two coders simultaneously coded one transcript and met to discuss their analytical approach for coding and come with a standardized approach for coding, i.e. granular capturing every concept, the coding that is a standalone statement that captures the essence of the expressed statement. Once in agreement, they both proceeded to analyze a few transcripts, then met again to develop the coding framework based on the research question. We had 3 broad questions and their definitions. They populated each broad question with the emerging codes.

1. Testing the reliability of the code

Then the two coders met with the research team to discuss the coding framework and determine its applicability to the raw information. The results were discussed and a few modifications were required before proceeding with the data analysis.

1. *Indexing* all study data against the framework

The codes for the remaining transcripts were indexed as per each element’s of the Kingdon’s multiple streams model. For example, once the statement is fitting a problem, then the statement is indexed under ‘problem’ and coded to capture the essence of the expressed statement.

1. *Charting* to summarize the indexed data

When all the indexing was completed, all the codes under each stream were reviewed. We combined those codes that have a central meaning to become a potential theme. We reflected on whether this potential has a central concept meaningful towards the question, coherent, how it relates to other findings, and whether it is a standalone theme or it can contain sub-themes. Finally, we provided a title for each theme.

1. *Mapping and interpretation* of patterns found within the charts

In this final step, we reviewed all the themes, sub-themes and quotes and looked for a compelling answer for the research question. We looked at what were the main factors forming the “problem”, the best practices forming the “policy” and the main actors and factors acting as “ political” influencers (Ritchie & Spencer, 1994).
